# Evidence for various interventions to reduce depressive symptoms for children and adolescents: protocol of a global evidence and gap map

**DOI:** 10.1186/s13643-025-02909-w

**Published:** 2025-08-08

**Authors:** Pengpeng Cao, Yuhao Li, Luyao Yang, Liping Guo, Jiyixi Hua, Shuo Zhang, Chuying Li, Bingxia Dang, Yanzhuang Sun, Ming Liu, Zheng Xu, Kehu Yang, Bei An

**Affiliations:** 1 Department of Pathogenic Biology, School of Basic Medical Sciences, Lanzhou University, Lanzhou, People’s Republic of China; 2 School of Philosophy and Sociology, Lanzhou University, Lanzhou, People’s Republic of China; 3 The Centre of Evidence-based Social Science, School of Basic Medicine, Lanzhou University, Lanzhou, People’s Republic of China; 4 The Centre of Evidence-based Social Science, School of Public Health, Lanzhou University, Lanzhou, People’s Republic of China

**Keywords:** Depressive symptoms, Children and adolescents, Evidence and gap map (EGM)

## Abstract

**Background:**

Children and adolescent depression conditions are responsible for high levels of disability burden and negatively influence academic participation and life-long outcomes while their interventions and outcomes remain controversial.

**Methods:**

We conducted a comprehensive search in multiple databases of primary studies. The EPPI‐Mapper mapping tool was applied to present identified studies as framework-described results.

**Discussion:**

This is the protocol for an evidence and gap map (EGM). The objectives of this EGM are to identify and map all randomized controlled studies (RCTs) on universal, school-based social and emotional learning programs for children and adolescents with depressive symptoms or diagnoses of depression, and to identify existing gaps in the evidence for creating an interactive, searchable, and publicly accessible EGM. The map will provide insights for researchers and decision-makers, build on the evidence bases in this field, and identify key areas for future research.

**Systematic review registration:**

Campbell Systematic Reviews cl2.20240091.

## Background

### Introduction

#### The problem, condition, or issue

Childhood and adolescence are unique stages of human development for laying foundations of health [1]. During these periods, children and adolescents’ brains are at critical stages of development with rapidly occurring physical, cognitive, and psychosocial growth, which affect their feeling, thinking, decision-making, and interaction with the world. Physical, emotional, and social changes make children and adolescents vulnerable to mental health issues [1]. Lots of research has shown that a transition from childhood or adolescence to adulthood is a critical period in the development of depression, with around half of adults with depressive disorders experiencing their first episode during childhood and adolescence [2–4].

The mental health of children and adolescents is a growing public health problem [5, 6]. Globally, one in seven children and adolescents are suffering from mental disorders, accounting for 13 percent of the global burden of disease in this population [7, 8]. Children and adolescents’ depression are recurring and chronic mental health problems that impair psychological functions and qualities of life [9]. In recent years, depression has increased in all age groups, but prevalences of children and adolescents have significantly surpassed those of adulthood [10]. Especially during the COVID-19 pandemic, children and adolescents face unprecedented challenges to their mental health status due to increasing risks of depression and anxiety [6, 11, 12]. Therefore, it is highly imperative to recognize, diagnose, and treat this disorder.

The demands for timely depression diagnoses and interventions in children and adolescents have been recognized as significant public health challenges. The current diagnosis of depression in children and adolescents is mainly based on the clinical assessment of the World Health Organization’s International Classification of Diseases and Diagnostic and Statistical Manual of Mental Disorders, Fifth Edition (DSM-V) [13, 14]. However, clinical presentations of depression in this population exhibit fundamental differences from adult depression, primarily attributing to ongoing cognitive development and the frequently non-specific nature of their symptoms.

Phenotypic expressions of depressive symptoms exhibit significant developmental variations, reflecting distinct neurocognitive maturation stages [15]. In preoperational children (2–7 years), depressive symptoms typically manifest predominantly through somatic complaints and behavioral dysregulations [16, 17]. Adolescents (≥ 12 years) present pronounced identity-related distress with increasing capacity for affective articulation [18]. Their various depressive emotions are often misinterpreted as teenage rebellion by their parents, such as anhedonia, lachrymose and irritability, hypophonia, and low self-esteem. They also exhibit a range of behavioral disorders, including hyperactivity, increased impulsivity, dislike or refusal to attend school, poor discipline in school, and strained relationships with parents, teachers, and peers, all of which may cause a significant decline in their overall functions [19].

### The interventions

A variety of interventions have been developed, including drug therapy, psychotherapy, physical exercise, and art therapy [20, 21]. Depression in children and adolescents is a mounting concern worldwide. In response, several countries and regions have implemented diverse measures to enhance the mental health of this vulnerable population. China has taken huge steps by issuing the “Action for a Healthy China—Action Program for the Mental Health of Children and Adolescents.” This program is designed to advance the implementation of relevant initiatives, including the Action for the Promotion of Health in Primary and Secondary Schools. Its overarching goal is to further reinforce efforts in addressing the mental health needs of children and adolescents, thereby promoting their mental well-being and fostering the holistic development of their overall quality [22]. The European Commission published the report “A systemic, whole-school approach to mental health and well-being in schools in the EU”, which provides a systematic, integrated framework to guide schools in effectively and sustainably promoting students’ mental health through a holistic, whole-school approach [23]. In addition, the World Health Organization (WHO) applies a variety of strategies, plans, and tools to assist governments in addressing the health needs of children and adolescents. For example, the “Helping adolescents thrive” initiative, a joint effort of WHO and United Nations International Children’s Emergency Fund (UNICEF), aims to strengthen policy and programming on adolescent mental health. The WHO regional office for the Eastern Mediterranean has developed a mental health training package for educators. It aims to raise awareness of the importance of mental health in schools and to guide the implementation of strategies to promote, protect, and restore students’ mental well-being [7].

The interventions can be classified as.

i) Pharmacological therapy: such as benzodiazepines [24], fluoxetine [25], vortioxetine [26], and alprazolam [27].
ii) Psychotherapy (offers to individuals, couples, families, or group members [28]. Common psychotherapeutic approaches include cognitive behavior therapy (CBT) [29–31], behavioral therapy [32, 33], behavioral activation [32, 34–36], interpersonal psychotherapy, psychodynamic therapy, and supportive therapy [37, 38].
iii) Complementary and alternative therapies, also known as complementary and alternative medicine (CAM), are a group of diverse medical and health care systems, practices, and products that are not typically considered part of conventional Western medicine. These may include practices such as traditional Chinese medicine, dietary supplements, physical activity, and other holistic approaches [28].
iv) Educational program and training: including psychological health education courses such as classroom lesson plans [39], school-based mindfulness interventions [40], training such as educational manuals [41].
v) Expressive therapies: expressive therapy is defined as the use of art, music, dance/movement, drama, poetry/creative writing, theatre, and sand tray in a psychotherapeutic, counseling, rehabilitation, or health care settings [42].
vi) Other interventions including digital health interventions [43], peer support [44], and other therapeutic approaches.

However, these interventions vary in terms of quantity, quality, types, settings, and target populations, which make the evidence challenging to navigate. In addition, identifying effective interventions for depression can inform strategies to reduce its potentially adverse effects on children and adolescents, improve their mental health, and promote a sense of well-being in their overall development.

We will create an evidence and gap map (EGM) of existing randomized controlled trials (RCTs), as described in the Campbell Guide to EGMs [45]. Specifically, we will develop an EGM focusing on RCTs that investigate interventions and outcomes related to depression in children and adolescents. The map will visualize the current landscape of RCTs on depression interventions and outcomes in the population to enhance their mental health, and inform future research.

### Why it is important to develop the EGM

Child and adolescent depression is a significant predictor of psychological problems in adulthood, and its persistence not only undermines an individual’s current mental, physical, and emotional well-being, but also has long-term consequences that extend into adulthood, potentially leading to increased challenges in future work, marital relationships, and overall life satisfaction [46, 47]. The increasing available evidence suggests that early diagnosis and treatment lead to a better prognosis [48, 49].

Early diagnosis and treatment are crucial for enhancing the effectiveness of depression interventions, preventing mental illness and relapse, and deepening our comprehension of individual well-being and whole quality of life in children and adolescents, including advocating a more holistic mental health promotion for child and adolescent depression and expanding the focus of the field to involve strategies that improve mental health. Given the widely varying effectiveness of interventions targeting depression in children and adolescents, there is an urgent need for a systematic approach to assess and compare different strategies.

This EGM will visualize available evidence on depression interventions for children and adolescents, highlight gaps in these interventions, and provide a common framework for mental health professionals, educators, policy-makers, and parents to discuss and develop strategies for addressing the complex needs of depressive symptoms or diagnoses in children and adolescents. It will also raise public awareness of the importance of early identification and intervention for depressive symptoms in children and adolescents, thereby contributing to improvements of young people’s well-being and reductions of the long-term impacts of depression on their lives.

#### Existing EGMs

We systematically searched several electronic databases including Cochrane, Campbell, EPPI, PubMed, and 3ie (International Initiative for Impact Evaluation). Currently, there are no existing EGMs or protocols that comprehensively cover all interventions for depression in children and adolescents, while there are six EGMs related to depression; two of them focus solely on single interventions for depression in children and adolescents:

- Yu et al. provided an EGM on the effectiveness of psychosocial support interventions for child and adolescent mental health, based on 448 primary studies and 249 systematic reviews, which focused on the mental health of children and adolescents in low- and middle-income countries (LMICs), to promote mental health and reduce or prevent mental health conditions in this population. The study found that research on psychosocial interventions for children and adolescents has predominantly been reactive rather than proactive, with most evidence focusing on addressing mental health conditions rather than prevention or mental health promotion [50].
- Campisi et al. created an EGM of micronutrients on depression in children (6–9 years) and adolescents (10–19 years) by reviewing 30 primary studies, which demonstrated that the most commonly studied micronutrients were vitamin D, zinc, iron, folate, or vitamin B-12 [51].

The remaining four EGMs or protocols focus on individuals with depression in other age groups, but two [52, 53] of these covered the groups of children and adolescents:

- Dsouza et al. planned to analyze systematic reviews, qualitative studies, and relevant working papers to develop a clear framework of intervention types and outcomes to outline the available evidence on interventions that can be used to improve the well-being of people with mental disorders. They planned to identify evidence gaps categorized by important intervention categories, regions, contexts, and population subgroups (Children (under 14 years of age), young adulthood (15–24), middle adulthood (25–44), and older adulthood (45–64) and elderly (65 + years old)) [53].
- Campbell et al. provided a mapping review and EGM on non-familial intergenerational interventions and their impacts on social and mental well-being of both younger individuals (under 30 years old) and older adults (aged 65 years and above). The EGM reviewed 500 research articles of any design relevant to intergenerational interventions. It aimed to provide decision-makers with an overview of the evidence base, enabling them to explore different interventions related to their population needs and the settings or resources available [52].
- Guo et al. planned to propose an EGM of available systematic reviews on the effectiveness of interventions for treating depression in adults (aged 18 and over) including psychotherapy, pharmacotherapy, and complementary and alternative therapies. They informed research commissioning and provided a scientific basis for developing healthcare policies and practices [21].
- Shang et al. planned to present an EGM on the effectiveness of non-pharmacologic interventions for treating depression in older adults (aged 60 years and above), for assessing the effectiveness and cost-effectiveness of non-pharmacologic interventions for the population to inform future research [54].

While four EGMs have examined depression interventions for children and adolescents to varying degrees, including two specifically focused on this population [50, 51] and the other two incorporated them within broader age groups [52, 53], which remained significant limitations in the current evidence landscape. Yu et al. [50] focused only on psychosocial interventions in LMICs, and Campisi et al. [51] only considered micronutrient supplementation, both of which ignored other key interventions like pharmacological treatments, behavioral therapies, and digital interventions. Furthermore, their analyses did not fully consider age-related differences in depression presentation. Therefore, it would be highly beneficial to develop an EGM encompassing all available interventions for children and adolescents’ depression. To achieve this, we will systematically search for RCTs that investigated interventions targeting depression or depressive symptoms in children and adolescents aged 0–18 years. This EGM will provide a comprehensive overview of the current evidence bases and identify gaps in research related to treating or preventing depression in this population.

To our knowledge, this is the first EGM to systematically map all intervention types—pharmacological, psychological, and complementary and alternative therapies—exclusively for children and adolescents (0–18 years) using evidence from RCTs. This method addresses gaps in prior EGMs that focused on a single intervention [50, 51] or broader age groups [52, 53]. Compared to existing EGMs, our study differs in two key aspects: (1) intervention comprehensiveness: While previous maps examined isolated approaches (e.g., micronutrients [51] or psychosocial support [50]), our EGM includes all major intervention categories. (2) Developmental specificity: we stratify analyses by critical neurodevelopmental stages (early childhood, pre-adolescence, adolescence), unlike previous broader age categorizations [52, 53].

## Objectives

Specifically, the objectives of the EGM are.

1. To create a methodological classification of interventions for depressive disorder and related outcomes;
2. To map existing RCTs on the prevention, promotion, and treatment of depressive disorder.

We hope to solve the following questions through this EGM:

1. What types of interventions for child and adolescent depression have been most frequently studied globally, and what critical gaps exist across different regions?
2. How are outcome measures distributed across various intervention approaches, and which intervention methods are most effective in achieving optimal outcomes?

## Methods

We will follow the Campbell Collaboration guidance for producing an evidence and gap map [45].

### Framework development and scope

The development of the frameworks was recognized as the most important and often difficult part of developing the evidence map. We finalized the categorization framework for programs relevant to intervention populations through stakeholder discussions and suggestions. It was further refined through piloting.

### Stakeholder engagement

We have formed an advisory group comprising methodologists, psychologists, psychiatrists, educational researchers, and experts in data collection and analysis, mental status assessment, and prevention of depressive disorders.

In February 2024, we invited several psychologists and psychiatrists (Lidong Wang, Zheng Xu, Kun Qiao, Qi Liu, and Qiangli Dong) to hold an initial meeting and gather feedback on the development of our EGM framework in the Centre for Evidence-Based Medicine at Lanzhou University in Gansu Province. During this meeting, we decided to focus on the classification of depressive disorders and relevant interventions. Our core teams (Pengpeng Cao, Yuhao Li, Bei An, Zheng Xu) planned to meet at least every other week to discuss the direction and scope of EGM.

### Conceptual framework

**Fig. 1:**
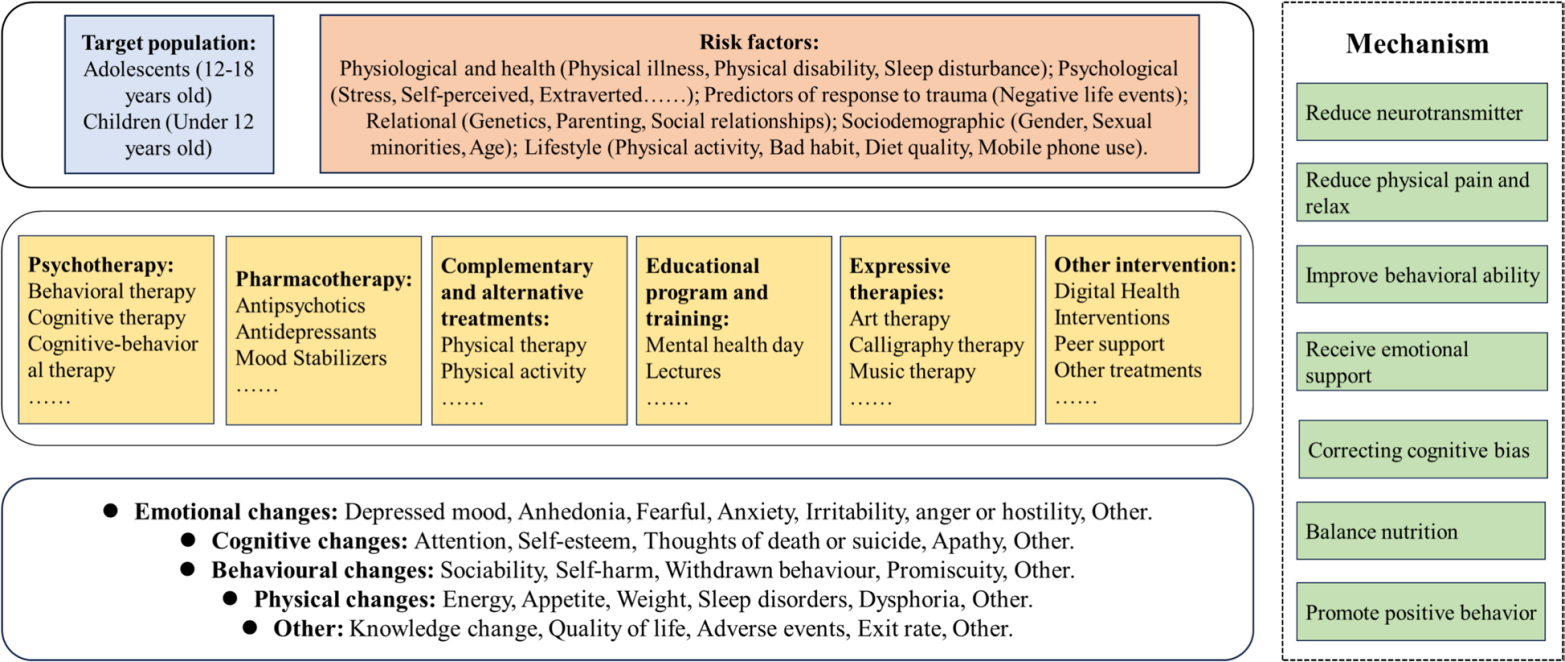
Conceptual framework for depression

The pathogenesis of depression is complex, stemming from a multifaceted interplay of social, psychological, and biological factors. The etiology often varies significantly among different patients, and even within the same individual, the contributing factors can differ across various stages of their lives or episodes of illness [55]. Many treatments have been proposed to target various causes of depression. For instance, medications improve mood by regulating neurotransmitters in the brain [56]. Psychotherapy alleviates emotional symptoms and improves behavioral coping by correcting patients’ cognitive biases [21]. Physical therapy, mainly through physical means, acts on the human body to regulate brain functions [57], thereby alleviating depressive symptoms. The conceptual framework of depression is shown below (Fig. 1).

### Dimensions

#### Types of study design

This map systematically summarizes interventions and outcomes relevant to depression in children and adolescents. We will only include RCTs of any interventions designed to treat, promote, or prevent the occurrence of depression or depressive symptoms in this population.

### Types of intervention/problem

Eligible studies must include interventions to treat, promote, or prevent depressive symptoms. The intervention-outcome framework identified six groups based on intervention types: psychotherapy, pharmacotherapy, educational programs and training, complementary and alternative therapies, expressive therapies, and other interventions. Each intervention type contains several sub-intervention types. Specific intervention categories are shown in Table 1. These will be further improved and developed through extensive piloting.

**Table 1:** Categories and subcategories of interventions

Categories	Sub-category	Example from included study
Psychotherapy	Behavioral therapy	[58]“To determine whether dialectical behavior therapy (DBT) for adolescents with bipolar spectrum disorder is more effective than standard of care (SOC) psychotherapy in decreasing suicide attempts over 1 year.”
	Cognitive therapy	[59] “A brief, group cognitive therapy prevention program can reduce the risk for depression in the adolescent offspring of parents with a history of depression.”
	Cognitive‐behavioral therapy	[60] “30 moderately depressed high school students were randomly assigned to either cognitive-behavioral treatment, relaxation training, or a wait-list control condition.”
	Interpersonal psychotherapy	[61] “The current study sought to examine whether interpersonal psychotherapy for depressed adolescents (IPT-A), an intervention that aims to treat depression by improving adolescents ‘attachment relationships, also has an impact on dysfunctional attitudes”
	Psycho‐dynamic psychotherapy	[62] “The aim of the present study was, according to the second protocol analysis, to analyze the moderating effect of personality disorders on treatment outcomes in depressed adolescents who received STPP with or without transference work.”
	Supportive psychotherapy	/
	Other psychotherapy	[63]“Towards this end, we aimed to design and preliminarily test an early psychotherapeutic intervention for preschool depression. In searching for early interventions applicable for adaptation to the treatment of preschool onset MDD, PCIT stood out as an appropriate and promising treatment modality”
Pharmacotherapy	Selective serotonin reuptake inhibitors (SSRIs)	[64]“This article is of a randomized, double-blind, placebo-controlled trial of fluoxetine in children and adolescents with depression.”
	Serotonin‐norepinephrine reuptake inhibitors (SNRIs)	[65]“The purpose of this study was to evaluate the efficacy and safety of duloxetine fixed dose in the treatment of children (7–11 years) and adolescents (12–17 years) with major depressive disorder (MDD).”
	Monoamine oxidase inhibitors (MAOIs)	/
	Tricyclic antidepressants (TCAs)	[66] “To determine amitriptyline's (AMI) efficacy in the acute treatment of adolescent major depressive disorder (MDD).”
	Norepinephrine and dopamine reuptake inhibitors (NDRIs)	/
	Serotonin antagonist and reuptake inhibitors (SARIs)	/
	Tetracyclic antidepressants (TeCAs)	/
	Other	[67]“We conducted an active, placebo-controlled trial to determine the safety and efficacy of intravenous esketamine in this population.”
Complementary and alternative treatments	Physical therapy	[68]“The present study examined the effect of a five-day TUS intervention on depression and anxiety symptoms in individuals with mild to moderate depression.”
	Physical activity	[69]“In this study, a randomized controlled trial was conducted to examine the effectiveness of adventure-based training in enhancing resilience and self-esteem and reducing depressive symptoms among juveniles.”
	Traditional Chinese Medicine	/
	Diet or supplement	[70]“Participants in the Diet Change group received the diet intervention instructions from our registered dietician via a 13-min video, available for re-watching online as needed.”
	Other	[71]“The aim of the study was to assess the effect of short time (6 weeks) bright light treatment (BLT) on depressive symptoms in female patients with the restrictive type of anorexia nervosa (AN-R).”
Educational program and training	Mental health day	/
	Mental health advocacy	[72]“In this indicated prevention trial, 341 at-risk youth were randomized to a group CB intervention, group supportive expressive intervention, CB bibliotherapy, or educational brochure control condition”
	Skills training	[73]“Two forms of short-term group therapy for depressed adolescents are compared. Adolescents were assigned to either a social skills training or therapeutic support group.”
	psychological health education course	[74] “OVK contains Cognitive Behavioral Therapy, social skills training, problem solving and decision making. Outcomes are measured at 6, 12, 18 and 24 months follow up, to monitor long term program effects.”
	Other	/
Expressive therapies	Art therapy	[75]“Thus, the present study aimed to evaluate the effectiveness of Pre-Texts arts-literacy intervention for adolescent depression and anxiety in Kenyan high school students, using a randomized controlled trial (RCT)”
	Calligraphy therapy	[76]“Those in the calligraphy group participated in 60-min calligraphy sessions for three weeks.”
	Horticultural therapy	/
	Music therapy	[77]“To determine the effectiveness of interactive media-based cognitive behaviour, art, and music therapies in treating depression in children who had been kidnapped in Nigeria, we used a randomized control trial approach”
	Drama therapy	/
	Dance/movement therapy	[78]“This study assessed the profiles of psychological health and changes in neurohormones of adolescents with mild depression after 12 weeks of dance movement therapy (DMT).”
	Poetry therapy and bibliotherapy	/
	Play therapy	[79]“The two-year intervention was a biweekly structured game led by a coach followed by critical reflection and discussion for 30 min.”
	Sand play therapy	/
	Integrated arts approach or intermodal (also known as multimodal) therapy	/
	Other	/
Other intervention	Digital Health Interventions	[80]“We aimed to evaluate the feasibility, acceptability, and potential impact of a theory-informed, co-designed digital intervention program, MoodHwb.”
	Peer support	[81] “On the basis of the proposed routine treatment, we conducted 8 weeks of model 328-based peer education intervention in the intervention group.”
	Other treatments	/

### Types of population

The subjects of this study were children and adolescents (under 18 years of age) with depression, depressive symptoms, or at potential risks of depression. If the study covers adults, children, and adolescents, information on children and adolescents will be extracted separately.

### Types of outcome measures

We divided outcome categories by analyzing clinical guidelines related to depression in children and adolescents, as well as categories used in DSM–V [20, 82]. The main outcome categories are shown in Table 2.

**Table 2:** Categories and subcategories of outcomes

Categories	Sub-category	Example from included study
Remission of depressive symptoms	Mild to moderate depressive disorder	[83]“The research team recruited adolescents ages 13–18 with major depressive disorder with moderate to severe symptom severity from outpatient psychiatric clinics, community primary care clinics, and a psychiatric inpatient unit between 2015 and 2018 in Rochester, Minnesota and the surrounding communities.”
	Major depressive disorder	[84]“Subjects were included who had a current episode of MDD of at least 2 months'duration, but not more than 2 years'duration.”
	Persistent depressive disorder	/
	Disruptive mood dysregulation disorder	/
	Premenstrual mood disorder	/
	Substance/drug induced depressive disorder	/
	Other specific depressive symptoms	/
	No specific depressive disorder	[61]“The study was approved and monitored by the site’s institutional review board. Adolescents were aged 12–17 with a Diagnostic and Statistical Manual of Mental Disorders, Fourth Edition, Text Revision (DSM-IV-TR) diagnosis of Major Depressive Disorder, Dysthymia, or Depressive Disorder Not Otherwise Specified (NOS)”
	Bipolar disorder	[58]“I This randomized clinical trial evaluates whether dialectical behavior therapy for adolescents with bipolar spectrum disorder is more effective than standard of care psychotherapy in decreasing suicide attempts over 1 year.”
	Unspecified	/
	Other	[85] “Sub-threshold depression (SD) has been associated with impairments in adolescent health which increase the rate of major depression.”
Emotional changes	Depressed mood	[86] “The 13-item Short Mood and Feelings Questionnaire assesses core symptoms of depression in children and adolescents.”
	Anhedonia	[77] “The CDI has 27 items with five dimensions that look at negative mood, ineffectiveness, interpersonal problems, lack of pleasure, and low self-esteem.”
	Fearful	/
	Anxiety	[87]“Negative affect was measured using the Depression Anxiety Stress Scale – Short form”
	Externalizing behavior	[88] “BYI encompasses 100 items, divided into 5 subscales that measure anxiety, depression, anger, disruptive behavior, and self-concept respectively.”
	Other	/
Cognitive changes	Attention	[85]“Mindful Attention and Awareness Scale (MAAS)”
	Self-esteem	[89] “We assessed self-esteem (how I think about myself questionnaire), anxiety (what I think and feel questionnaire), depression (short mood and feelings questionnaire), and antisocial behaviour (behaviour and activities checklist).”
	Thoughts of death or suicide	[90]“Outcomes were based on blinded independent evaluator assessments at week 6 (randomization) and every 6 weeks during continuation treatment (6, 12, 18, 24, and 30), and included measures of symptoms and functioning (CDRS-R, Suicide Severity Rating Scale-Short, CGI) and course of illness using the Adolescent-Longitudinal Interval Follow-Up Evaluation (A-LIFE)”
	Apathy	/
	Other	/
Behavioural changes	Sociability	[80] “Standardized questionnaires were used to explore changes in depression literacy and stigma, help-seeking behavior, self-efficacy, behavioral activation, depression and anxiety symptoms, and general behavior”
	Self-harm	[91] “Outcomes included mood symptoms, suicidal ideation and behavior, nonsuicidal self-injurious behavior, and emotional dysregulation.”
	Withdrawn behaviour	/
	Promiscuity	/
Physical changes	Energy	[92]“We administered the 4-item energy/fatigue subscale of the RAND-36 instrument that assesses health-related quality of life in several domains.”
	Appetite	/
	Weight	[87]“The weight and shape subscales form two of the four subscales assessed by the Eating Disorder Examination-Questionnaire, and are considered to best represent the broad construct of weight concerns that has been found to be one of the strongest risk factors for disordered eating in adolescents”
	Sleep disorders	[92]“TST is defined as actual time slept, not including time attempting to fall asleep, middle of the night awakenings, and time awake while in bed at the end of the sleep period.”
	Dysphoria	/
	Other	/
Other	Knowledge improving of mental health	[80]“Secondary outcomes included the potential effect on mood, knowledge, attitudes, and behavior after using the program”
	Quality of life	[93]“Results from the present analysis indicated significant impairment in quality of life (QoL) among patients in an acute episode of pediatric bipolar depression.”
	Adverse events	[84]“There were some AEs that were observed more frequently in STS-treated subjects. These included decreased appetite (3.3% vs. 1.3%), agitation (2.6% vs. 1.9%), anxiety (2.6% vs. 1.3%), insomnia (5.9% vs. 2.6%), somnolence (4.6% vs. 2.6%), upper respiratory tract infection (7.2% vs. 2.6%), and vomiting (4.6% vs. 2.6%).”
	Exit rate	/
	Other	/

The intervention and outcome categories will be piloted when preparing the protocol.

### Other eligibility criteria

Studies will not be limited by geographic location. Only studies published in English or Chinese will be included. There is no restriction on the time of publication.

### Types of settings

We will include interventions in any setting, i.e., participants receiving treatment in a range of settings (hospital, community, school, home, etc.). We will code settings so that evidence can be filtered by settings.

### Search methods and sources

The Information Specialist will design and test a draft search strategy for MEDLINE via Ovid SP. The strategy will be presented to the review team and advisory board for their feedback and modifications. The final search approach and selection of databases will be jointly determined by the review team. The agreed-upon strategy will be adapted for other databases, and the final searches will be executed.

Searching databases: we will search a wide range of bibliographic databases and websites to identify all relevant research:

- MEDLINE
- Embase
- PsycINFO

Search strategy:

**Table:** 

Database	Search strategies
Medline	**1** exp Depression/ **2** Depressive Disorder/ **3** (depress* or dysthymi*).tw **4** (adjustment disorder* or affective disorder* or affective symptom*).mp **5** exp Anxiety/ **6** exp Anxiety Disorders/ **7** (agoraphobi* or anxiety or anxio* or phobi* or panic or obsessi* or compulsi* or OCD or GAD or PTSD or posttrauma* or post trauma* or post trauma* or stress disorder or neurosis or neuroses or neurotic or psychoneuro*).tw **8** 1 or 2 or 3 or 4 or 5 or 6 or 7 **9** exp Infant, Newborn/ **10** exp Infant, Premature/ **11** exp Infant/ **12** exp Infant Behavior/ **13** exp Child/ **14** exp Child, Preschool/ **15** exp Child Behavior/ **16** exp Child Development/ **17** exp Pediatrics/ **18** exp Puberty/ **19** exp Adolescent/ **20** exp Adolescent Behavior/ **21** exp Adolescent Development/ **22** exp Young Adult/ **23** exp Schools, Nursery/ **24** exp Child Day Care Centers/ **25** exp Child Care/ **26** exp Education, Graduate/ **27** exp Universities/ **28** exp Students/ **29** exp Schools/ **30** 9 or 10 or 11 or 12 or 13 or 14 or 15 or 16 or 17 or 18 or 19 or 20 or 21 or 22 or 23 or 24 or 25 or 26 or 27 or 28 or 29 **31** (preterm* or prematur* or postmatur* or perinat* or postnat* or neonat* or newborn* or new born or infan* or baby* or babies or toddler* or preschool* or child* or pediat* or paediat* or kid or kids or prepubescen* or prepuberty* or puberty or pubescen* or teen* or young* or youth* or minors* or under ag* or underag* or juvenile* or girl* or boy* or preadolesc* or adolesc* or nursery or prekindergarten or kindergarten* or early childhood education or preschool* or elementary education or elementary school* or primary education or primary school* or K 12* or K12 or 1 st grade* or first grade* or grade 1 or grade one or 2nd grade* or second grade* or grade 2 or grade two or 3rd grade* or third grade* or grade 3 or grade three or 4th grade* or fourth grade* or grade 4 or grade four or 5th grade* or fifth grade* or grade 5 or grade five or 6th grade* or sixth grade* or grade 6 or grade six or intermediate general or middle school* or secondary education or secondary school* or 7th grade* or seventh grade* or grade 7 or grade seven or 8th grade* or eight grade* or grade 8 or grade eight or 9th grade* or ninth grade* or grade 9 or grade nine or 10th grade* or tenth grade* or grade 10 or grade ten or 11th grade* or eleventh grade* or grade 11 or grade eleven or 12th grade* or twelfth grade* or grade 12 or grade twelve or junior high* or highschool* or high school* or preuniversity or pre university or college* or undergrad* or tertiary education or tertiary school* or postsecondary education or postsecondary school* or universit* or prevocational or vocational or class room* or curricul* or education* or learner* or lesson* or pupil* or school* or student*).ab,ti **32** 30 or 31 **33** 8 and 32 **34** randomized controlled trial.pt **35** controlled clinical trial.pt **36** randomi#ed.ti,ab **37** randomly.ab **38** sham.ab **39** trial.ab **40** groups.ab **41** (control$ adj3 (trial or study)).ab,ti **42** ((singl$ or doubl$ or tripl$ or trebl$) adj3 (blind$ or mask$ or dummy)).mp **43** 34 or 35 or 36 or 37 or 38 or 39 or 40 or 41 or 42 **44** (animals not (humans and animals)).sh **45** 43 not 44 **46** 33 and 45
Embase	**1** exp depression/ **2** (depress* or dysthymi*).mp **3** (adjustment disorder* or affective disorder* or affective symptom* or Depressive Disorder*).mp **4** exp anxiety/ **5** exp anxiety disorder/ **6** (agoraphobi* or anxiety or anxio* or phobi* or panic or obsessi* or compulsi* or OCD or GAD or PTSD or posttrauma* or post trauma* or post trauma* or stress disorder or neurosis or neuroses or neurotic or psychoneuro*).mp **7** 1 or 2 or 3 or 4 or 5 or 6 **8** exp infant/ **9** exp newborn/ **10** exp prematurity/ **11** exp child behavior/ **12** exp child/ **13** exp preschool child/ **14** exp child behavior/ **15** exp child development/ **16** exp pediatrics/ **17** exp puberty/ **18** exp adolescent/ **19** exp adolescent behavior/ **20** exp adolescent development/ **21** exp young adult/ **22** exp nursery school/ **23** exp child day care/ **24** exp child care/ **25** exp graduate education/ **26** exp university/ **27** exp student/ **28** exp school/ **29** (preterm* or prematur* or postmatur* or perinat* or postnat* or neonat* or newborn* or new born or infan* or baby* or babies or toddler* or preschool* or child* or pediat* or paediat* or kid or kids or prepubescen* or prepuberty* or puberty or pubescen* or teen* or young* or youth* or minors* or under ag* or underag* or juvenile* or girl* or boy* or preadolesc* or adolesc* or nursery or prekindergarten or kindergarten* or early childhood education or preschool* or elementary education or elementary school* or primary education or primary school* or K 12* or K12 or 1 st grade* or first grade* or grade 1 or grade one or 2nd grade* or second grade* or grade 2 or grade two or 3rd grade* or third grade* or grade 3 or grade three or 4th grade* or fourth grade* or grade 4 or grade four or 5th grade* or fifth grade* or grade 5 or grade five or 6th grade* or sixth grade* or grade 6 or grade six or intermediate general or middle school* or secondary education or secondary school* or 7th grade* or seventh grade* or grade 7 or grade seven or 8th grade* or eight grade* or grade 8 or grade eight or 9th grade* or ninth grade* or grade 9 or grade nine or 10th grade* or tenth grade* or grade 10 or grade ten or 11th grade* or eleventh grade* or grade 11 or grade eleven or 12th grade* or twelfth grade* or grade 12 or grade twelve or junior high* or highschool* or high school* or preuniversity or pre university or college* or undergrad* or tertiary education or tertiary school* or postsecondary education or postsecondary school* or universit* or prevocational or vocational or class room* or curricul* or education* or learner* or lesson* or pupil* or school* or student*).mp **30** 8 or 9 or 10 or 11 or 12 or 13 or 14 or 15 or 16 or 17 or 18 or 19 or 20 or 21 or 22 or 23 or 24 or 25 or 26 or 27 or 28 or 29 **31** 7 and 30 **32** randomized controlled trial.ti,ab **33** controlled clinical trial.ti,ab **34** randomi#ed.ab **35** randomly.ab **36** sham.ab **37** trial.ab **38** groups.ab **39** (control$ adj3 (trial or study)).ab,ti **40** ((singl$ or doubl$ or tripl$ or trebl$) adj3 (blind$ or mask$ or dummy)).mp **41** 32 or 33 or 34 or 35 or 36 or 37 or 38 or 39 or 40 **42** (animals not (humans and animals)).mp **43** 41 not 42 **44** 31 and 43
PsycINFO	**1** exp Major Depression/ **2** exp"Depression (Emotion)"/ **3** exp Bipolar Disorder/ **4** exp Affective Disorders/ **5** (depress* or dysthymi*).mp **6** (adjustment disorder* or affective disorder* or affective symptom*).mp **7** exp Anxiety/ **8** exp Anxiety Disorders/ **9** exp Climate Anxiety/ **10** exp Computer Anxiety/ **11** exp Generalized Anxiety Disorder/ **12** exp Health Anxiety/ **13** exp Separation Anxiety/ **14** exp Separation Anxiety Disorder/ **15** exp Social Anxiety/ **16** (agoraphobi* or anxiety or anxio* or phobi* or panic or obsessi* or compulsi* or OCD or GAD or PTSD or posttrauma* or post trauma* or post trauma* or stress disorder or neurosis or neuroses or neurotic or psychoneuro*).mp **17** 1 or 2 or 3 or 4 or 5 or 6 or 7 or 8 or 9 or 10 or 11 or 12 or 13 or 14 or 15 or 16 **18** exp Infant Development/ **19** exp Neonatal Period/ **20** exp Neonatal Development/ **21** exp Premature Birth/ **22** exp Child Behavior/ **23** exp Child Health/ **24** exp Child Psychiatry/ **25** exp Child Psychology/ **26** exp Child Psychopathology/ **27** exp Child Support/ **28** exp Preschool Students/ **29** exp Pediatrics/ **30** exp Puberty/ **31** exp Adolescent Behavior/ **32** exp Adolescent Development/ **33** exp Adolescent Health/ **34** exp Schools/ **35** exp Boarding Schools/ **36** exp High Schools/ **37** exp Junior High Schools/ **38** exp Middle Schools/ **39** exp Nursery Schools/ **40** exp Child Day Care/ **41** exp Day Care Centers/ **42** exp Childhood Development/ **43** exp Child Care/ **44** exp Early Childhood Development/ **45** exp Preschool Students/ **46** exp Graduate Students/ **47** exp Higher Education/ **48** exp Graduate Education/ **49** exp College Students/ **50** exp Colleges/ **51** exp Students/ **52** 18 or 19 or 20 or 21 or 22 or 23 or 24 or 25 or 26 or 27 or 28 or 29 or 30 or 31 or 32 or 33 or 34 or 35 or 36 or 37 or 38 or 39 or 40 or 41 or 42 or 43 or 44 or 45 or 46 or 47 or 48 or 49 or 50 or 51 **53** (preterm* or prematur* or postmatur* or perinat* or postnat* or neonat* or newborn* or new born or infan* or baby* or babies or toddler* or preschool* or child* or pediat* or paediat* or kid or kids or prepubescen* or prepuberty* or puberty or pubescen* or teen* or young* or youth* or minors* or under ag* or underag* or juvenile* or girl* or boy* or preadolesc* or adolesc* or nursery or prekindergarten or kindergarten* or early childhood education or preschool* or elementary education or elementary school* or primary education or primary school* or K 12* or K12 or 1 st grade* or first grade* or grade 1 or grade one or 2nd grade* or second grade* or grade 2 or grade two or 3rd grade* or third grade* or grade 3 or grade three or 4th grade* or fourth grade* or grade 4 or grade four or 5th grade* or fifth grade* or grade 5 or grade five or 6th grade* or sixth grade* or grade 6 or grade six or intermediate general or middle school* or secondary education or secondary school* or 7th grade* or seventh grade* or grade 7 or grade seven or 8th grade* or eight grade* or grade 8 or grade eight or 9th grade* or ninth grade* or grade 9 or grade nine or 10th grade* or tenth grade* or grade 10 or grade ten or 11th grade* or eleventh grade* or grade 11 or grade eleven or 12th grade* or twelfth grade* or grade 12 or grade twelve or junior high* or highschool* or high school* or preuniversity or pre university or college* or undergrad* or tertiary education or tertiary school* or postsecondary education or postsecondary school* or universit* or prevocational or vocational or class room* or curricul* or education* or learner* or lesson* or pupil* or school* or student*).mp **54** 52 or 53 **55** 17 and 54 **56** exp Randomized Controlled Trials/ **57** exp Clinical Trials/ **58** randomi#ed.ti,ab **59** randomly.ab **60** sham.ab **61** trial.ab **62** groups.ab **63** (control$ adj3 (trial or study)).ab,ti **64** ((singl$ or doubl$ or tripl$ or trebl$) adj3 (blind$ or mask$ or dummy)).mp **65** 56 or 57 or 58 or 59 or 60 or 61 or 62 or 63 or 64 **66** (animals not (humans and animals)).sh **67** 65 not 66 **68** 55 and 67

### Analysis and presentation

#### Report structure

The EGM report will encompass the subsequent components: executive summary, background, interventions, results, and conclusions. The executive summary will summarize the report for future policy planning and research. The background section will delve into the contemporary landscape of adolescent and childhood depression, exploring its profound societal implications and multifaceted challenges it poses. In addition, we will state the objectives of this EGM and describe its scope by defining the intervention and outcome framework.

The methodology section will describe the following components: data sources and retrieval methods, inclusion and exclusion criteria, study screening process, data extraction methods, and quality assessment methods. Additionally, this section will focus on elaborating the search strategy, including specific restrictions and filters used, while adhering to the Preferred Reporting Items for Systematic Reviews and Meta-Analyses (PRISMA) guidelines [94].

The results section will present the number, type, and quality of studies retrieved for each intervention and outcome category. Additionally, this section will feature an interactive EGM to provide a graphical representation of the available evidence.

In the final section of the report, we will discuss the potential impacts of the EGM on researchers, policy-makers, and other key stakeholders. We anticipate that the findings will provide valuable insights for researchers and policy-makers, enhance the existing evidence bases in the field, and identify key areas for future research.

In addition, the report will include the following tables and figures:

Figure: PRISMA flowchart.

Figure: EGM-Primary Research (non-interactive).

Figure: Online interactive EGM-with filters.

Table: number of studies by intervention and subcategory.

Table: number of studies by population.

Table: number of studies by intervention category and study confidence.

### Filters for presentation

The EGM will have two primary dimensions: interventions (rows) and outcomes (columns).

Additional dimensions (to be used as filters) will be.

1. Targeted populations: gender (girl/female, boy/male and other), age (children and adolescents);
2. Types of intervention: psychotherapy, pharmacotherapy, complementary and alternative therapies, educational program and training, expressive therapies, and other interventions;
3. Country: any country involved;
4. Region: East Asia & Pacific; Europe & Central Asia; Latin America & Caribbean; Middle East & North Africa; North America; South Asia; Sub-Saharan Africa;
5. Country classifications by income level: (low, lower-middle, upper-middle, and high income classified by New World Bank country based on income level (https://datahelpdesk.worldbank.org/knowledgebase/articles/906519-world-bank-country-and-lending-groups))
6. Setting for the intervention: community, school, hospital, home, and other;
7. Disease severity: mild, moderate, major, severe, extremely severe, and unspecified;
8. Measure of depression and depressive symptoms: structural, semi-structural interview, and unstructured interview;
9. Period of symptoms: > 12 months, 6 months–12 months, 1 month–6 months, < 1 month, and not state;
10. Confidence of evidence: low quality, middle quality, and high quality evaluated by Risk of Bias 2 [95].
11. Conflict of interest: yes, no, and unclear; 
12. Intervention group: individual-based, group-based, peer support, and self-help;
13. Intervention mode: online, offline, and mixed intervention;
14. Intervention implementors: teacher, psychological counsellor, social worker, nurse, doctor, self-help, and others.
15. Intervention duration: > 9 months, 4–9 months, < 4 months, and unclear;
16. Follow-up time: > 12 months, 6–12 months, 3–6 months, < 3 months, and not state.
17. Physical condition/intervention type: health/prevent and unhealth/treatment.
18. Medical history: naïve medicine or historical medicine.

In the hard copy of the EGM (the evidence gap and map report), multiple 2 × 2 representations of the EGM will be reported with different dimensions as row and column headings. A copy of the coding form will be included as an annex to the EGM protocol and report.

In the online version of the map, the additional dimensions may be used as filters. The online version will include references with brief summaries of each study based on the plain language.

### Dependency

The units of analysis for this EGM are the included RCTs. If the same study were published in multiple papers, we only included the most recent version. If papers from the same study contain different outcome indicators, these papers will be used solely to report different outcomes.

### Data collection and analysis

#### Screening and study selection

Initial screening will be done by two independent authors through title and abstract based on population, type of study. Studies that could not be identified would be decided by a third author. The initial studies were re-screened by reviewing the full text to finalize the included studies. We will present a PRISMA flowchart to demonstrate the process of study selection.

### Data extraction and management

The data extraction form will be developed by two reviewers to define the map framework and variables to be extracted. It will be pilot-tested on 20 studies, with selected variables recorded in an Excel file. The two reviewers will independently extract and graph the data, discuss the results, and iteratively update the data extraction form throughout the process.

Data will be extracted from all included studies using a standardized data extraction and coding form. Four researchers will independently perform the coding using the EPPI-Reviewer software (a software used for managing and encoding data, https://eppi.ioe.ac.uk/cms/Default.aspx?tabid=2914). In the event of disagreements, a fifth researcher will mediate, and a final decision will be made through a group discussion involving all five researchers. Coding will be based on the intervention and outcome framework, and will include additional filter content such as demographic information, intervention type, study quality, intervention site, conflict of interest, duration of intervention, and follow-up time.

### Tools for assessing risk of bias/study quality of included reviews

The reliability of the findings in the included systematic evaluations will be assessed using the Systematic Evaluation of Measurement Tools Assessment (ROB2.0) [95]. The risk of bias in included studies was assessed independently by four investigators (Pengpeng Cao, Yuhao Li, Luyao Yang, and Jiyixi Hua). The investigators have received systematic training to ensure the consistency of the results. Any discrepancies were resolved by consensus within the review team. The assessment included (1) the randomization process; (2) deviations from the intended interventions; (3) missing outcome data; (4) measurement of the outcome; (5) selection of reported results; (6) overall bias. For every included study, each of these items was classified as “low risk of bias” (“ +"), “high risk of bias” (“-"), or “unclear risk of bias” (“?").

### Methods for mapping

We will use the EPPI mapper to perform related tasks, such as literature screening and data extraction, and generate an interactive EGM. It is a tool that generates and visualizes maps as interactive HTML files in a Web browser. This map was generated using the data exported from EPPI-Reviewer. All the data can be openly accessed on this file. To ensure long-term accessibility, data will be simultaneously archived in the EPPI mapper and updated every 5 years.

Prior to coding, coders were uniformly trained and 20 randomly selected included studies were used as a pilot to “revise, refine and define” the framework. The pilot exercise included the actual coders. Cleaning of coded data after coding was completed included identifying uncoded data, out-of-range codes and investigating unexpected patterns in the data, as well as examining a random sample of coded records and systematically checking all records for any codes that appeared to have been incorrectly used by coders.

## Discussion

An EGM is a systematic evidence synthesis product that shows the available evidence relevant to a specific research question. The scope of an EGM is usually broader than that of a systematic review [45]. EGMs are applied to identify gaps that need to be filled with new evidence, to collect research findings for review, and to improve the discovery and use of research by policy-makers, research commissioners, and researchers. It is also used to generate reviews of higher-level evidence products such as guidelines [45]. This EGM would provide an overview of the available evidence on interventions for depression in children and adolescents. This EGM will present existing evidence distributions through an interactive matrix (interventions × outcomes), serving as an evidence base for (1) clinical decision support systems (incorporating symptom severity-intervention matching algorithms and developmental stage-specific adaptation protocols); (2) policy development toolkits (including regional evidence gap heatmaps and cost-effectiveness analysis modules); and (3) implementation guidelines for diverse settings (healthcare facilities, schools, and communities). The research outcomes will directly inform updates to clinical practice guidelines, optimization of public health resource allocation, and global mental health policy formulation, thereby bridging the gap between scientific evidence and real-world clinical practice and health policy decision-making.

## Plans for updating the EGM

After completion, we will update it every five years. The first author and corresponding author will be responsible for updating the EGM.
